# Army Medical Appointments

**Published:** 1857-06

**Authors:** 


*Army Medical Appointments.*—At a recent meeting of the Army Medical Board, held in New York city, they report to the War Department the names of Robert Bartholomew, Mo., Joseph C. Bailey, Pa., J. Cooper McKee, New York, W. A. Carswell, S. C., and Kirtley Ryland, Mo., as having been examined and found qualified for appointment in the Medical Staff.

We are rejoiced to see the name of our young friend, Dr. Kirtley Ryland as one of the successful candidates. Our acquaintance and association with Dr. R. during his connection with the Medical Department of the Iowa State University, as Demonstrator of Anatomy, warrants us in saying that his professional talent and untiring energy render him peculiarly qualified for the position to which he has been appointed. He has our best wishes for future success.

